# DEET‐cyclodextrin inclusion complexes for mosquito and tick repellency: Extended repellency time and no zebrafish embryotoxicity

**DOI:** 10.1002/ps.71001

**Published:** 2026-06-11

**Authors:** Gessyka Rayana Silva Pereira, Gleidson Cardoso, Maria Luiza Kuhn Camargo, Michele Resende Machado, Mayara Macedo Barrozo, Juscelino Rodrigues, Gisele Augusto Rodrigues de Oliveira, Caio Monteiro, Christian Luz, Ricardo Neves Marreto, Stephânia Fleury Taveira

**Affiliations:** ^1^ Laboratory of Nanosystems and Drug Delivery Devices (NanoSYS), School of Pharmacy, Universidade Federal de Goiás Setor Leste Universitário Goiânia Brazil; ^2^ Environmental Toxicology Research Laboratory, School of Pharmacy, Universidade Federal de Goiás Goiânia Brazil; ^3^ Biology, Ecology and Tick Control Laboratory, Veterinary and Animal Science School Universidade Federal de Goiás Goiânia Brazil; ^4^ Invertebrate Pathology Laboratory, Instituto de Patologia Tropical e Saúde Pública, Universidade Federal de Goiás Goiânia Brazil

**Keywords:** HPβCD–DEET complex, slow release, repellent formulation, vector, topical administration

## Abstract

**BACKGROUND:**

Repellent formulations are a cost‐effective strategy for protection against disease vectors. *N*,*N*‐diethyl‐meta‐toluamide (DEET) is widely used to repel mosquitoes and ticks, including *Amblyomma sculptum*, the vector of *Rickettsia rickettsii* (the agent of Brazilian spotted fever), and *Aedes aegypti*, responsible for dengue, zika, and chikungunya. This study aimed to develop cyclodextrin (CD)–DEET complexes for topical application. Phase‐solubility studies identified the most suitable CD, and hydroxypropyl‐β‐cyclodextrin (HPβCD)–DEET was selected for further investigation. *In vitro* release and skin permeation were evaluated. Embryotoxicity was assessed in a zebrafish model, and repellent efficacy was investigated against both species.

**RESULTS:**

Phase‐solubility studies revealed A_L_‐type profiles for αCD, γCD, and HPβCD, with slopes between 1 and 2, and K_2:1_ values of 4738, 4494, and 2169, respectively. HPβCD–DEET exhibited slower DEET release compared to the control (26.7% vs 50.4% over 10 h, *P* < 0.05). Although DEET permeation to the receptor medium was similar (*P* > 0.05), HPβCD reduced DEET retention in the stratum corneum by 2.6‐fold (*P* < 0.05). HPβCD–DEET did not cause embryotoxicity in permeated DEET concentrations (69 mg L^−1^). Repellency bioassays showed HPβCD–DEET efficacy against *A. sculptum* for 168 h (80.5–100%) and full protection against *A. aegypti* for 7 h (>95%).

**CONCLUSION:**

These results highlight the potential of HPβCD–DEET complexes for safer and effective repellent formulations. © 2026 The Author(s). *Pest Management Science* published by John Wiley & Sons Ltd on behalf of Society of Chemical Industry.

## Graphical abstract

Hydroxypropyl‐β‐cyclodextrin (HPβCD)–*N*,*N*‐diethyl‐meta‐toluamide (DEET) complex extended tick repellency to 168 h. Full protection against *Aedes aegypti* for 7 h with HPβCD‐DEET. HPβCD reduced DEET release and skin retention significantly. No embryotoxicity observed below 325 mg L^−1^ in zebrafish model. HPβCD‐DEET offers a safer, long‐lasting topical repellent option.

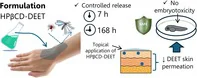

**ABBREVIATIONS**
αCD: alpha cyclodextrin
βCD: beta cyclodextrin
CD: cyclodextrin
DEET: (N,N‐diethyl‐meta‐toluamide)
γCD: gamma cyclodextrin
HPβCD: hydroxypropyl‐β‐cyclodextrin
RS: remaining skin
Kc: stability constant
SC: stratum corneum

## 1 INTRODUCTION

Vector‐borne pathogens, which necessitate the involvement of blood‐sucking vectors to transmit disease‐transmitting organisms,^1^ are not just illnesses but a global health crisis. These diseases, prevalent and affecting millions yearly,^2^ persist as a significant cause of morbidity and mortality worldwide today,^3^ demanding immediate attention and action.

Mosquitoes and ticks, recognized as the primary carriers of several diseases, pose a significant threat to public health. For instance, *Aedes aegypti* is the culprit behind the rapid spread of dengue, yellow fever, zika, and chikungunya, the most common arboviruses in recent years.^4^, ^5^ Similarly, *Amblyomma sculptum*, a species from the *Amblyomma cajennense* complex, is the primary vector of the bacterium *Rickettsia rickettsii*, causing Brazilian spotted fever, a febrile and acute disease with high lethality in humans.^6^, ^7^

**Figure 1:**
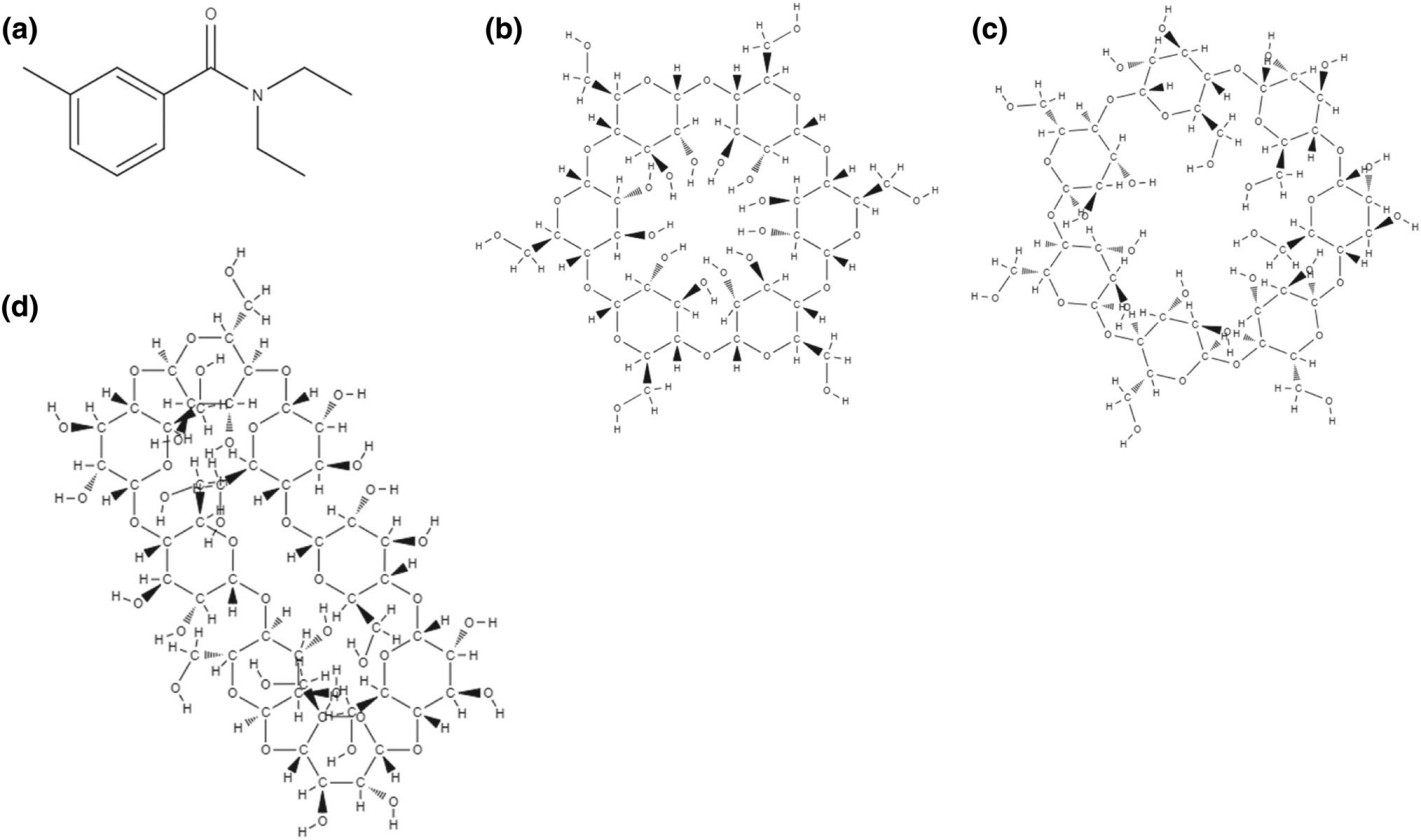
Chemical structure of (a) DEET, molecular weight 191.3 g mol^−1^, log *P* 2.02, water solubility 1.68 mg mL^−1^ and vapor pressure of 0.002 mmHg at 25 °C,^9^ (b) alpha‐cyclodextrin (αCD), molecular weight 972.8 g mol^−1^ and water solubility of 145 mg mL^−1^ at 25 °C, (c) beta‐cyclodextrin (βCD), molecular weight 1135 g mol^−1^ and water solubility of 18.6 mg mL^−1^ at 25 °C and (d) gamma‐cyclodextrin (γCD), molecular weight 1297.1 g mol^−1^ and water solubility of 232 mg mL^−1^ at 25 °C.^10^

Preventive measures against insect bites are crucial due to the high incidence of diseases. Topical repellents are commonly used for this purpose. These repellents contain volatile substances that repel arthropods by inducing them to move away from the repellent. *N*,*N*‐diethyl‐meta‐toluamide (DEET) (Fig. 1(a)) is a synthetic active ingredient commonly used to repel mosquitoes.^8^ It is the oldest and the most powerful repellent available in the market, thus it is the reference standard.^8^ Studies have shown it is also effective against *A. sculptum* nymphs, providing over 90% repellency in laboratory and field conditions.^3^

Despite its promising results, DEET (Fig. 1(a)) is a highly hydrophobic molecule, which makes it challenging to dissolve in water‐based formulations. To enhance its solubility, commercial insect‐repellent formulations containing DEET often include ethanol, isopropanol, or propylene glycol. However, high concentrations of ethanol, when applied regularly, could lead to carcinogenic effects or be absorbed through the skin, resulting in toxic levels, especially in children.^11^ Additionally, these solvents can act as penetration enhancers, disrupting the stratum corneum (SC) membrane and promoting DEET penetration and its systemic absorption.^12^ Therefore, the concentration of DEET allowed for babies and children is limited, and the number of daily applications is restricted in some countries, such as Brazil.^13^ Hence, safer formulations with little or no ethanol are important to minimize these aspects.

Different drug delivery systems have been proposed to improve the solubilization of DEET in water‐based formulations, reduce or eliminate organic solvents in formulations, and regulate the release rate of the active ingredient. The studies have proposed polymeric and polysaccharide microcapsules,^14^, ^15^, ^16^, ^17^ calcium alginate beads,^18^ solid lipid nano and microparticles,^19^, ^20^, ^21^ fibers,^22^ and micellar systems with DEET.^23^ However, low load capacity and yield, use of organic solvents, high energy costs of production, and laborious methods are common issues.^24^

The use of cyclodextrins (CDs) (Fig. 1(b)–(d)) to control the volatility of actives is a compelling strategy for obtaining repellent formulations. Surprisingly, this approach has been relatively underexplored for DEET topical delivery. CDs are cyclic oligosaccharides obtained from starch degradation and are generally recognized as safe.^25^, ^26^ They resemble hollow truncated cones (Fig. 1(b)–(d)) with a hydrophilic surface and an inner hydrophobic cavity, enabling interaction with hydrophobic molecules. Additionally, chemical modifications in the CD surface, such as hydroxypropylation, methylation, and acetylation, improved their water solubility.^26^ Thus, we hypothesized that DEET complexation with CDs could enhance its solubility, potentially eliminating the need for organic solvents in the formulations. Furthermore, CD–DEET complexes could control the active release, prolonging the formulation effect and reducing DEET skin permeation.

While a few studies have explored the use of CDs in DEET formulations for repellent delivery,^27^, ^28^, ^29^, ^30^, ^31^, ^32^ several key areas still require further investigation. For instance, Proniuk and coworkers^29^ prepared different emulsions, substituting ethanol for up to 20% of γCD or 30% hydroxypropyl‐β‐cyclodextrin (HPβCD). Their formulations with CDs decreased DEET release, but the effectiveness in repelling mosquitos was not confirmed. Nagai and coworkers^30^ proposed formulations with DEET, ethanol, and HPβCD for a better‐controlled release, low skin permeation, and formulation stickiness. They achieved some of these goals, but they still used organic solvents. The gaps in the cited research include the lack of evaluation of the effectiveness of the developed formulations in different arthropod models, lack of concern about DEET skin permeation or the choice of CD, the presence of solvents or non‐eco‐friendly methods for obtaining the complexes, and the absence of safety studies in representative models.

Thus, the current scenario highlights the need for a simple repellent system that is easy to obtain, effective, and safe without organic solvents or laborious methods. Therefore, this study aimed to develop complexes of CD and DEET to obtain an effective repellent formulation with a sustained release of the active ingredient and low toxicity for use. For this, phase solubility studies were carried out to choose the CD based on greater solubilization. Then, *in vitro* release, and permeation studies were carried out to evaluate the behavior of DEET through the formulation for topical application. In addition, for the first time, toxicity studies with the zebrafish model were performed for the developed formulations, and repellency bioassays were performed to verify their effectiveness against ticks and mosquito species that are relevant to public health.

## 2 MATERIALS AND METHODS

### 2.1 Materials

*N*,*N*‐Diethyl‐meta‐toluamide (DEET) (≥ 97%) and 3,4‐dichloroaniline reagent were purchased from Sigma Aldrich® (St. Louis, USA). Alpha cyclodextrin (αCD; Cavamax W6®, lot 601 086, 973 Da), beta cyclodextrin (βCD; Cavamax W7®, lot 701 134, 1135 Da), gamma cyclodextrin (γCD; Cavamax W8®, lot 801 152, 1297 Da), and hydroxypropyl‐β‐cyclodextrin (HPβCD; Cavitron W7 HP7®, lot A1411A0050, 1520 Da, molar substitution 1.03 as estimated by ^1^H NMR, degree of substitution 7.21) were kindly donated by Ashland Inc. (São Paulo, SP, Brazil). High‐performance liquid chromatography (HPLC) grade acetonitrile and methanol were obtained from JT Baker (Phillipsburg, NJ, USA). Water was purified using a Milli‐Q Millipore Simplicity (Bedford, MA, USA) with a 0.22 μm pore end filter.

### 2.2 Analytical procedure

DEET was quantified by spectrophotometry and HPLC with ultraviolet (UV) detection. The methods were validated following the International Guide for Harmonization of Technical Requirements for Registration of Pharmaceutical Products for Human Use (ICH).^33^ DEET amount in formulations and release experiments was determined at 240 nm using a UV/visible (Vis) spectrophotometer (Thermo Scientific, Waltham, MA, USA). A linear calibration curve was obtained (*y* = 0.0264*x* + 0.0062, *r* = 1) from 3 to 30 μg mL^−1^ using a mixture of methanol:water (50:50, v/v) as diluent. The limit of quantitation was 0.5 μg mL^−1^ and the detection limit was 0.16 μg mL^−1^. Accuracy and precision were satisfactory (values presented relative standard deviation and error less than 5%). Selectivity was investigated for formulation components and receptor medium, and no interference was observed at 240 nm.

HPLC method was developed according to the literature^34^ and validated as recommended by the ICH^33^ to quantify DEET in different skin layers. Briefly, the HPLC system was an Agilent 1260 Infinity II with a quaternary pump (G7111B), automatic injection system (G7129A), and UV–Vis detector (G7114A) (Agilent Technologies, Santa Clara, CA, USA). A ZORBAX® SB‐C18 column (4.6 × 250 mm, 5 μm) was used. The wavelength was 254 nm, the injection volume was 20 μL, and the flow was 1.0 mL min^−1^. The mobile phase comprised acetonitrile:water (50:50, v/v), and the analysis time was 10 min. Data were processed using Open Lab software. A linear calibration curve (*y* = 119 729*x* + 33 949, *r* ^2^ = 0.9996) was obtained over the 1–30 μg mL^−1^ concentration range. Accuracy and precision were satisfactory (values presented relative standard deviation and error less than 5%). The selectivity (formulations and skin layers) was investigated, and no interference was observed in the DEET retention time.

### 2.3 Phase solubility studies

The shake flask method was used to determine the DEET solubility, and phase solubility diagrams were prepared according to the Higuchi and Connors^35^ method. Aqueous solutions containing 0.001–0.01 M of natural CDs (α, β, and γCD) and semi‐synthetic CD (HPβCD) were prepared (*n* ≥ 3), and DEET was added in excess. Samples were kept under orbital shaking for 48 h at 300 rpm and 25 °C to reach equilibrium. Solubility of DEET in the absence of CDs was also determined. Then, samples were centrifuged at 3.200 × g for 30 min, and uncomplexed DEET (supernatant oil) was discharged. The complexed sample (clear aqueous solution) was appropriately diluted in methanol:water (1:1, v/v), and DEET content was determined spectrophotometrically (Thermo Scientific, Waltham, MA, USA). The stability constant of the formed complex (*Kc*) was calculated. For slope values less than 1, *Kc* was determined according to Eqn (1). For slope values between 1 and 2, *Kc* was calculated based on Eqn (2).^36^

(1) $${\mathrm{Kc}}_{1:1}=\frac{\text{slope}}{S_{0}\;\left(1-\text{slope}\right)}$$

(2) $${\mathrm{Kc}}_{2:1}=\frac{\text{slope}}{S_{0}2\;\left(2-\text{slope}\right)}$$

where *S*_0_ is the DEET solubility in methanol–water mixture without CD.

### 2.4 Preparation of HPβCD‐DEET complexes

The selection of the CD–DEET complexes was based on the results from phase solubility studies (section 2.3). HPβCD–DEET complexes were obtained by dispersing DEET (8%, w/w) in aqueous solutions of HPβCD (60%, w/w), 1:1 M ratio, under magnetic stirring at 250 rpm at 25 °C, covered with paraffin plastic film (Parafilm M®, PM996, Neenah, WI, USA) to avoid DEET evaporation. After complete solubilization, formulations were stoked in amber glass vials and sealed with a stopper. DEET content was determined spectrophotometrically (section 2.2), and pH analyses were performed using a pH meter (PG1800, Gehaka, São Paulo, SP, Brazil).

### 2.5 *In vitro* DEET release

DEET release was evaluated using a modified Franz‐type diffusion cell (Unividros, São Paulo, Brazil) and a dialysis membrane (MWCO 1 kD; Spectrum Labs, Rancho Dominguez, CA, USA) placed horizontally between the donor and the receptor chamber. PBS buffer solution (pH 7.4) was used as receptor medium (15 mL) to ensure sink conditions. The system was kept under magnetic stirring at 300 rpm and 37.0 ± 0.5 °C. Then 650 mg of HPβCD–DEET was added at the donor compartment (8% of DEET), and the experiment was performed for 10 h (*n* = 6). Next, 0.5 mL of the medium was collected from the receptor compartment at 2, 4, 6, 8, and 10 h. Equal volumes of fresh receptor medium were replaced. To minimize the loss of DEET by evaporation, the openings of the Franz diffusion cells were sealed with paraffin plastic film (Parafilm M®, PM996, Neenah, WI, USA). The amount of DEET released was determined spectrophotometrically (section 2.2). The control formulation was 8% DEET dissolved in an ethanol:water solution (60:40, v/v). The kinetic models applied are shown in Eqns ((3), (4), (5))–((3), (4), (5)).

(3) $$F=F_{0}+k_{0}t\;\left(\text{zero}-\text{order}\;\mathrm{eq}.\right)$$

(4) lnF=lnF0−K1t2.303first−ordereq.

(5) $$F={k\;H\;}^{\frac{1}{2}}\;\left(\text{Higuchi},\;,\text{eq},.\right)$$

where *F* represents the fraction of drug released over time (*t*), *F*_0_ is the initial amount of drug in solution, and *k*_0_, *k*1, and *kH* are the apparent rate constants for each equation.^37^ The flow (*J*) was calculated from the curve with the highest slope value.

### 2.6 Skin permeation studies

A skin permeation study followed the same experimental conditions as the release studies (section 2.5). However, a biological membrane (full‐thickness porcine ear skin) was used as a model for human skin.^38^ Unscalded porcine ear skin was obtained from Sol Nascente slaughterhouse (Goiânia, GO, Brazil) (CNPJ 73.918.757/0001–31), properly regulated for the local health surveillance department. Full‐thickness porcine ear skin was placed between the donor and receptor compartment, with the stratum corneum (SC) facing the donor compartment. At 5, 7, 9, 11, and 24 h, 0.5 mL of the receptor medium was collected and replaced with fresh medium. The amount of DEET permeated was determined by the HPLC method (section 2.2).

#### 2.6.1 Skin uptake

After permeation studies, the skin was removed from the diffusion cell, washed with distilled water, and placed on a smooth surface with the SC facing upwards. The area of the skin where the diffusion occurred was submitted to the tape‐stripping technique^38^ to separate SC from the remaining skin (RS). The RS was cut into small pieces. To extract DEET, tape strips with SC and the RS processed were placed separately in individual tubes and immersed in 5 mL of acetonitrile:water (1:1). The mixture was homogenized with a vortex (Vortex AP56, Phoenix Luferco, Araraquara, SP, Brazil) for 2 min. Then, samples were ultrasonicated for 20 min. After 2 h of rest, the samples were filtered with a 0.45 μm polyvinylidene difluoride membrane and analyzed by HPLC (section 2.2).

### 2.7 Zebrafish embryo‐larval toxicity assessment

#### 2.7.1 Zebrafish husbandry and egg collection

Adult male and female zebrafish (*Danio rerio*) between 3 and 6 months of age were housed at the EnvTox [Environmental Toxicology Research Laboratory, Federal University of Goias (UFG)]. The maintenance procedures and experimental protocols were approved by the Ethics Committee for Animal Use at the UFG (CEUA, institutional certificate No. 072/2020). The animals' health and well‐being were monitored daily during all experimental phases according to guidelines from the National Council for the Control of Animal Experimentation (CONCEA).

The animals were kept in separate tanks in a rack Hydrus system (Alesco, Monte Mor, SP, Brazil) with a recirculating system using water filtered by reverse osmosis, a highly effective water filtration process with activated carbon and biological filters and disinfected by UV light. The system maintained a constant temperature of 26 ± 1 °C, pH at 7.5 ± 0.5, electrical conductivity at 0.7 mS cm^−1^ ± 1.0, dissolved oxygen at 8 ppm, and a light/dark cycle of 12 h. The nitrate, nitrite, and ammonia levels were monitored regularly. This water from the animal maintenance system was used to prepare the test HPβCD–DEET formulation.

Adult organisms were fed commercial dry flake food (ColorBits Tetra®, São Paulo, SP, Brazil) and live brine shrimp (*Artemia salina* RN®, Natal, RN, Brazil). For the collection of eggs, adult fish at a ratio of 2:1 (male:female) were placed together prior to the onset of darkness on the day before the test. On the day of the test, zebrafish eggs were collected approximately 30 min after natural mating, transferred to Petri dishes containing reconstituted water, rinsed in distilled water to remove impurities, and examined under a stereomicroscope (Leica S9i, Heerbrugg, Switzerland).

Unfertilized or damaged eggs were discarded following the guidelines of CONCEA. The success of fertilization was checked, and only batches of eggs with a minimum fertilization rate of 90% were employed. Viable (fertilized) eggs were selected and used in the fish embryo acute toxicity (FET) test as OECD recommend.^39^

#### 2.7.2 FET test

The FET test was performed according to OECD Test Guideline 236.^39^ Twenty fertilized eggs per concentration were randomly selected and carefully distributed in a 24‐well plate filled with 2 mL of DEET (14.5, 31.5, 69, 150, and 325 mg L^−1^), HPβCD (108.5, 236.25, 517.5, 1125, and 2437.5 mg L^−1^) and HPβCD‐DEET formulation in the following concentrations (C1–C5): 108.5 mg L^−1^ HPβCD + 14.5 mg L^−1^ DEET (C1), 236.25 mg L^−1^ HPβCD + 31.5 mg L^−1^ DEET (C2), 517.5 mg L^−1^ HPβCD + 69 mg L^−1^ DEET (C3), 1125 mg L^−1^ HPβCD + 150 mg L^−1^ DEET (C4), 2437.5 mg L^−1^ HPβCD + 325 mg L^−1^ DEET (C5). The selection of DEET concentrations was based on the results of *in vitro* permeation studies using HPβCD–DEET (section 2.6). The HPβCD concentration levels were taken into consideration to determine the median concentration (LC_50_).

Maintenance water was utilized as a negative control (NC) and internal plate controls, Dimethyl sulfoxide ‐ DMSO (0.01% only for DEET) as a solvent control (SolC) and 3,4‐dichloroaniline at 4 mg L^−1^ as a positive control. The dissolved oxygen concentration, pH, and conductivity in the NC and highest test concentration of the test compound were measured at the beginning and termination of the test.

The test was conducted in triplicate in a climatized chamber at 26 °C, 12 h light for 96 h. Neither food nor aeration was provided during exposure. Lethal (egg coagulation, no somite formation, non‐detachment of the tail from the yolk sac, and no heart beating) and sublethal (effects on the eye and body pigmentation, absorption of the yolk sac, hatching rate, swimming bladder inflation, presence of edemas, spine and tail deformities) effects were determined at 24, 48, 72, and 96 h post‐fertilization employing a stereomicroscope (Leica S9i, Wetzlar, Germany), according to Kimmel and coworkers^40^ and Nagel.^41^

### 2.8 Repellency tests

#### 2.8.1 Ticks

*A. sculptum* nymphs were used in repellence laboratory bioassays. They were obtained using a method adapted from Sanavria and Prata.^42^ Briefly, adult females (teleogenic) were collected from naturally infested cattle of the Faculty of Veterinary at UFG and deposited in cell culture plates that were stored in a Biochemical Oxygen Demand (BOD) incubator under controlled temperature and humidity conditions (27 ± 1 °C and 80 ± 10%, respectively) for the oviposition period.

Following oviposition, the eggs were stored under the same controlled conditions in 5‐mL plastic syringes, sealed with dry cotton pads. This approach was crucial for obtaining the larvae (around 35 days). The engorgement of the larvae was then performed using the technique of inoculation in capsules,^43^ applied to the shaved back of New Zealand rabbits (Ethics Committee on the Use of Animals: UFG 053/2021). These rabbits were kept in individual cages with food and water *ad libitum*, under controlled temperature (20 °C). After approximately 4 days, the engorged larvae were collected from the rabbits and stored in syringes, as mentioned above, until reaching the nymph stage (approximately 15 days). Unfed nymphs aged 20–25 days were then used in our laboratory bioassays for repellency.

#### 2.8.2 Mosquitoes

The *A. aegypti* colony was kept at the Invertebrate Pathology Laboratory of the Institute of Tropical Pathology and Public Health, Goiânia, Brazil. Adult males and females were reared in screened cages (50 × 40 × 50 cm) at 25 ± 5 °C, 75 ± 10% relative humidity (RH), natural photophase and fed *ad libitum* with a 10% sucrose solution.^44^ Adult females were fed mouse blood (Ethics Committee on the Use of Animals: UFG 06/2021). Amber glass containers (100 mL) containing filter paper (90 mm in diameter) and 80 mL of distilled water were placed inside the cages, where the females laid their eggs. Twice a week the filter papers containing eggs were removed and then placed in a moist chamber (RH > 98%) for 2 days to complete embryogenesis, after which they were stored in a plastic box (12 × 26 × 33 cm) at 25 ± 5 °C and 75 ± 10% RH until use. To produce females used in the bioassays, filter papers containing embryonated eggs were transferred to plastic basins (29 × 15 cm) containing around 1 L of tap water heated to 35 °C. After hatching, the larvae were fed with crushed cat food (Bom Preço®, Salto de Pirapora, SP, Brazil) until pupation, and then transferred to disposable plastic cups (50 mL) containing 30 mL of tap water and placed in a covered container (500 mL). Females aged between 24 and 48 h after emergence were used in the repellency bioassay.

#### 2.8.3 Repellence bioassays

##### 2.8.3.1 Repellent activity against *A. sculptum* nymphs

The Petri dish bioassay was performed as described by Bissinger and coworkers^45^ and Barrozo and coworkers.^7^ The assays were performed in an environment with controlled temperature (27 ± 2 °C) and humidity (70 ± 10%). Glass Petri dishes (total area = 63.6 cm^2^) containing two semicircles (individual area = 31.8 cm^2^) of filter paper (Whatman qualitative, cat no. 1001–90 mm) were used (Supporting Information, Fig. S1). The left side of the plate received the treatment to be analyzed and the right side the respective control. Each semicircle received 250 mg (7.86 mg cm^−2^) of sample. A control trial was performed, and the paper semicircles did not contain any treatment. After drying the filter papers (around 15 min), six non‐engorged *A. sculptum* nymphs were placed in the central region of each plate. Each plate was then closed with strong, fine‐meshed, transparent fabric and secured with an elastic band. The displacement of *A. sculptum* nymphs was evaluated at times of 1, 3, 5, 10, 15, 30, 60, 120, and 240 min and 24, 48, 72, 96, and 168 h after the beginning of each experiment. In each analysis, ticks that remained in the untreated semicircle were considered repelled. The mean percentage of repellency corresponded to the percentage of ticks on the control side of the Petri dish. The study was carried out in sextuplicate. In the control group, a homogeneous distribution between the two sides of the Petri dish was expected.

##### 2.8.3.2 *In vivo* repellent activity against *A. aegypti* females

The repellency test was conducted in strict adherence to the WHO protocol,^46^ approved by the UFG Ethical Committee (CAAE 51073021.5.0000.5083). The test was performed in screened metal cages (30 × 20 × 20 cm) containing 36 adult females (5 days).^46^ Two cages with mosquitoes were assigned to each participant, one for testing the HPβCD–DEET and another for the negative control (CD aqueous solution). A 3 × 10‐cm area on each volunteer's forearm was delimitated using a plastic stopper and a rubber glove for mosquito exposure (Supporting Information, Fig. S2), and 0.3 g of the formulation and the negative control were applied to each exposure area. After 30 min of application, each treated forearm was placed in a different cage for 3 min. The number of mosquito landings and bites was recorded at each time point, and the test lasted 8 h. The repellency percentage was calculated using Eqn (6).

(6) $$\text{Repellency percentage}\;\left(\%\right)=\frac{NB-\mathrm{NT}}{NB}\times 100$$

where NB represents the number of bites or landings on the control sample and NT represent those numbers on the test substance.

### 2.9 Statistical analysis

The results were expressed as the mean ± standard deviation (SD). Statistical analyses were performed with the GraphPad Prism 5.03 software (GraphPad Inc., San Diego, CA, USA) for pH, *in vitro* release, and permeation studies and significant differences between the groups were determined using Student's *t*‐test or ANOVA followed by Tukey's multiple comparison test, with *P* < 0.05 as the minimum level of significance. Repellence laboratory bioassays were performed with the chi‐square goodness of fit test (Quantpsy.org), with *P* < 0.05 as the minimum significance level. For the zebrafish model, the significance of the differences was also calculated using one‐way ANOVA followed by Dunnett's *post hoc* test. Each experimental value was compared with its corresponding control. Data were expressed as the mean ± standard deviation, and statistical significance was set at *P* < 0.05. Toxicity was expressed as median effective (EC_50_) and lethal (LC_50_) concentrations with 95% confidence limits.

## 3 RESULTS AND DISCUSSION

### 3.1 Phase solubility studies

**Figure 2:**
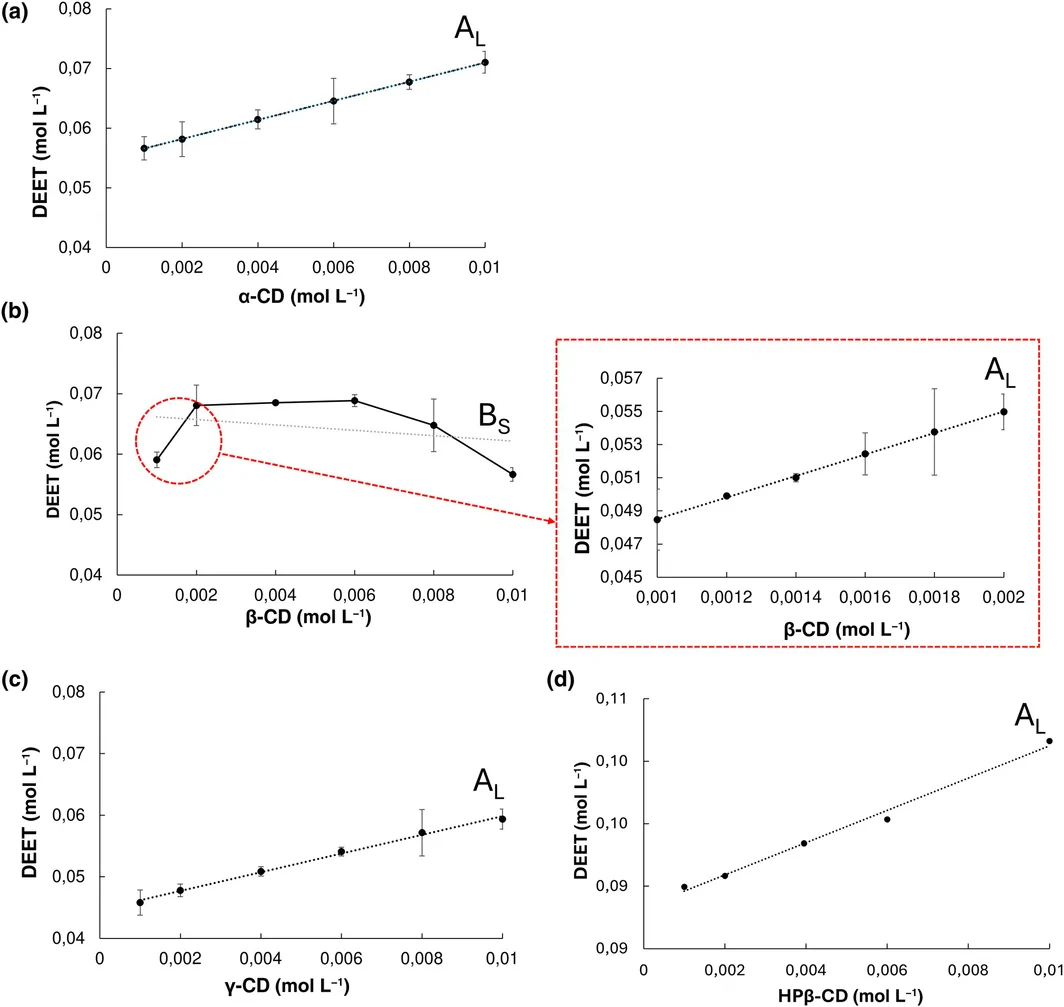
Phase‐solubility diagrams of the complexes: (a) DEET and αCD, (b) DEET and βCD, with the ascendent portion (0.001–0.002 mol L^−1^) highlighted in the red square, (c) DEET and γCD and (d) DEET and HPβCD.

Phase solubility diagrams of CDs were obtained (Fig. 2). Considering the different solubilities of CDs,^26^ aqueous solutions containing 0.001–0.01 M of CDs were obtained for α and γCDs (Fig. 2(a),(c)). They gave rise to an A_L_‐linear‐type solubility curve in which the increase in DEET solubility occurred with the rise in concentration of CDs.^35^ βCD has the lowest aqueous solubility and thus presented the B_S_ type curve (Fig. 2(b)). This curve exhibited an initial increase in solubility, followed by a plateau and a subsequent drop, possibly due to the formation of insoluble complexes that precipitate.^36^ Lower concentrations of βCD were also evaluated, i.e., concentrations that covered the ascending part of the B_S_ curve (0.001 to 0.002 mol L^−1^) (Fig. 2(b), red square).

The formation of the complexes can influence the solubilities of both the guest molecule and the CD.^35^, ^47^ According to Higuchi and Connors,^35^ the A_L_‐type diagrams indicate that the overall solubility of a substance increases with rising CD concentrations due to the creation of soluble complexes. For guest molecule:CD 1:1 ratio, the slope of a linear plot is less than one, and *Kc* is calculated as demonstrated in Eqn (1), but if the slope is greater than 1 unity and less than 2, the complex formed is likely to be a second order or higher, with respect to the drug, but first‐order with respect do CD, being determined as demonstrated by Eqn (2).^47^ In fact, A_L_‐type diagrams of αCD and γCD demonstrated slopes between 1 and 2, with values of 1.6 and 1.5, respectively. In this case, *K*_2:1_ (2:1, drug: CD ratio) was achieved to be 4738 and 4494 M^−1^, respectively. While these considerations are helpful, it is important to note that CDs can self‐associate on surfaces of active molecules, promoting solubilization by non‐inclusion interactions, and thus stoichiometry could be affected by its determination in phase solubility studies. Additionally, higher order systems are characterized by stepwise binding constants, i.e., formation of complexes 1:1 with one additional molecule. This may explain why CDs of such different sizes can produce similar constants. In the case of αCD, it seems that 1:1 complex could associate with one additional DEET molecule. Conversely, for γCD, the larger cavity may allow for the inclusion of two DEET molecules. For βCD, the *Kc* was not calculated from the Bs‐type curve.

The solubility of DEET in HPβCD demonstrated an A_L_‐type phase‐solubility diagram (Fig. 2(d)). The slope was also between 1 and 2 (1.3), resulting in a *K*_2:1_ (2:1, drug: CD ratio) of 2169 (Eqn (2)), which is lower than the *Kc* found for αCD and γCD. Thus, DEET seems to have a weaker interaction with HPβCD compared to αCD and γCD. However, this could be favorable for DEET release and its repellent activity. Additionally, HPβCD demonstrated the best DEET solubilization capacity, allowing DEET to be used at concentrations similar to those in commercial products containing organic solvents. Therefore, HPβCD was selected for subsequent studies.

#### 3.1.1 Obtention of HPβCD–DEET complexes

The complexes were obtained with a high recovery rate (99.60 ± 0.72%). The pH was 6.74 ± 0.03, suitable for topical delivery. The systems produced were transparent, fluid, and consisted of a single homogeneous phase. Notably, the developed system exhibited low viscosity, facilitating application and making it suitable for spray packaging, which is simple and easy to store. In contrast, other systems, such as repellent emulsions, can form large droplets, complicating the spraying process. Since HPβCD is highly soluble in water (approximately 30× more soluble than βCD), and DEET, although chemically characterized as an oil, appears as a liquid, this facilitates the creation of a fluid system that is easy to use.

### 3.2 *In vitro* release and permeation studies

#### 3.2.1 *In vitro* release study

**Figure 3:**
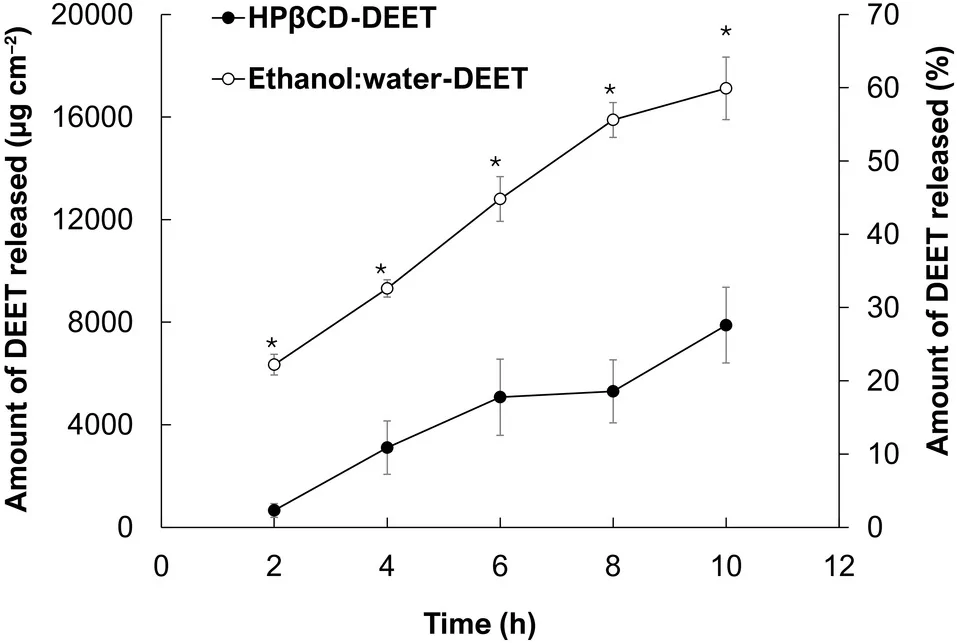
DEET *in vitro* release study from different formulations: ethanol:water–DEET solution (60:40, v/v) and HPβCD–DEET aqueous complex over 10 h of experiment in Franz‐type diffusion cells (*n* = 6). The receptor media was comprised of PBS buffer solution (pH 7.4). *Indicates differences between formulations (*P* < 0.05).

HPβCD complexes were selected to assess the impact of CD on the *in vitro* release and permeation of DEET. The control formulation (ethanol:water solution) released approximately 24% of DEET in the first 2 h (Fig. 3). In contrast, the HPβCD–DEET released only about 2% of the DEET during the same period, which is roughly 10 times less. By the end of the 10‐h study, the control released 50.4% of DEET, whereas the complexes released only 26.7%. Throughout the analysis, the amount of DEET released was significantly lower in the HPβCD complexes compared to the control (*P* < 0.05). Moreover, the release flux was substantially lower for HPβCD‐DEET complexes (3977.3 ± 408.7 μg cm^−2^ h^−1^) compared to the control solution (6494.7 ± 842.5 7 μg cm^−2^ h^−1^) (*P* < 0.05).

To date, the literature contains only one study that has evaluated the release rate of HPβCD–DEET complexes. Proniuk and coworkers^29^ used 20% DEET and 30% HPβCD with the goal of developing a repellent formulation. The authors demonstrated that HPβCD significantly reduced DEET release, which is consistent with the results of our study. They also found that HPβCD concentrations below 30% did not provide satisfactory release control. Despite these findings, no comprehensive research has yet evaluated the formulations' permeation, release, and repellency. This highlights the urgent need for further systematic studies in this area.

#### 3.2.2 *In vitro* permeation study

**Figure 4:**
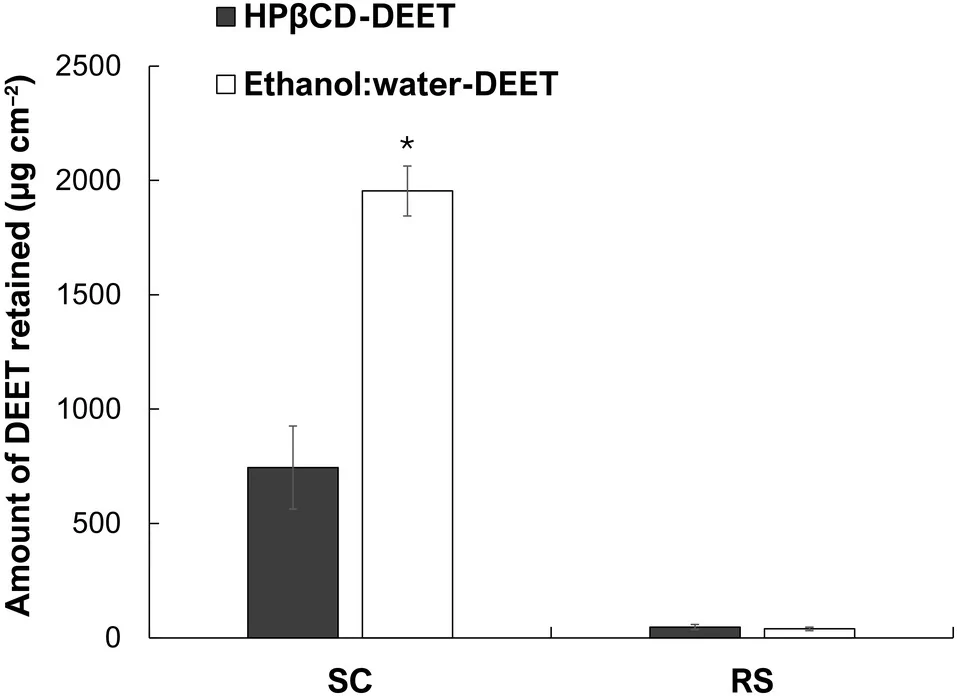
Amount of DEET retained in the stratum corneum (SC) and remaining skin (RS) after topical administration of HPβCD–DEET and control formulation (ethanol:water–DEET) for 24 h.

The amount of DEET permeated into the receptor solution from the control formulation (ethanol:water–DEET) and HPβCD–DEET was similar (*P* > 0.05), resulting in 553.8 ± 70.2 μg cm^−2^ of DEET (or 69 mg L^−1^) in the receptor medium when HPβCD–DEET was topically applied. Despite these findings, it was evident at the end of the 24‐h period that the control formulation volatilized rapidly, leading to a reduction in the initial formulation volume in the donor compartment, even with the occlusion of this compartment with parafilm. Nonetheless, the ethanol present in this formulation led to increased retention of DEET in the SC, as demonstrated in Fig. 4. This accumulation of DEET in the SC may act as a reservoir, facilitating its partitioning into deeper skin layers. In contrast, the HPβCD–DEET formulation prevented the formation of this reservoir, resulting in a decrease of 2.6‐fold of DEET permeation in this layer (*P* < 0.05).

The use of CD complexes reduces DEET permeation and may help minimize or eliminate side effects, which limit its use in children younger than 2 years and in pregnant women up to the sixth gestational month.^8^ These toxic effects are mainly associated with the use of organic solvents in formulations, which favor the absorption of these molecules through the skin, resulting in potentially harmful systemic effects.^48^, ^49^ In addition, DEET cutaneous permeation is also linked to the loss of repellent efficacy, as the compound penetrates the skin and leaves the surface, where it should remain to exert its repellent action against mosquitoes.^48^ Also, DEET volatilization can be rapid, leading to insufficient protection over extended periods.

Generally, the short repellency time is always related to skin absorption, evaporation, and rub‐off or wash‐off. Skin permeation is the main reason for product loss of effectiveness.^17^ Reducing skin permeation can keep more DEET available on the skin surface and increase the effectiveness of a repellent product.

### 3.3 Embryotoxicity study

We investigated the embryotoxicity of the HPβCD–DEET complex and its individual components (DEET and HPβCD) using zebrafish early‐life stage as a model organism, evaluating both lethal and sublethal endpoints. The concentration ranges applied in this formulation toxicity study were defined based on DEET skin permeation to the receptor chamber of vertical diffusion cells (section 3.3). For instance, in 24 h of the study, about 69 mg L^−1^ (or 553.8 ± 70.2 μg cm^−2^) of DEET permeated to the receptor medium. This concentration was calculated by converting the amount per area to concentration in the receptor volume using the formula (553.8 μg cm^−2^ × 1.87 cm^−2^)/15 mL = 69.04 μg mL^−1^, corresponding to approximately 69 mg L^−1^.

Thus, zebrafish embryos were exposed to different concentrations of the HPβCD, DEET and HPβCD–DEET complex. Lethal (LC_50_) and sublethal (EC_50_) effects in embryos and larvae were evaluated each 24 h until 96 h.

A wide range of concentration of HPβCD (108.5 mg L^−1^ up to 2437.5 mg L^−1^) caused no significant lethality in zebrafish embryo and larvae. The mortality rate ranged from 3.34% to 6.67% at 96 h of exposure, therefore it was not possible to determine the LC₅₀ value for HPβCD–DEET complex (Table 1; LC₅₀ > 2437.5 mg L^−1^). Similarly, no significant sublethal effects, such as pericardial or yolk sac edema, spine or head deformities, were observed, even at the highest concentration of HPβCD tested. The sublethal effect rate ranged from 1.7% to 2.2% after 96 h of exposure (Table 2 and Fig. 6), confirming that HPβCD did not induce embryotoxicity. Accordingly, no statistically significant adverse effects, whether lethal or sublethal, were observed, and the NOEC was established at 2437.5 mg L^−1^ (Table 1).

**Table 1:** Median lethal concentration (LC_50_), median effective concentration (EC_50_), lowest observed effect concentrations (LOEC), and no observed effect concentrations (NOEC) for mortality and malformations in zebrafish early‐ life stage after 96 h of exposure to HPβCD, DEET and HPβCD–DEET formulation.

Endpoints (mg L^−1^)	HPβCD	DEET^a^	HPβCD–DEET
LC_50_	>2437.5	201.6 (158.0–257.1)	—
EC_50_	—	—	—
LOEC	—	325	2437.5 mg L^−1^ HPβCD + 325 mg L^−1^ DEET (C5)
NOEC	2437.5	150	1125 mg L^−1^ HPβCD + 150 mg L^−1^ DEET (C4)

^a^ Confidence interval shown in parentheses.

—, not determined.

**Table 2:** The low frequency abnormalities induced by HPβCD, DEET, and HPβCD–DEET after 96 h of exposure.

Group: HPβCD						
Concentration (mg L^−1^)		2437.5	1125	517.5	236.25	108.5
Sublethality parameters	Pericardial edema	2.2%	0%	0%	0%	0%
	Yolk sac edema	2.2%	0%	0%	0%	0%
	Spine deformity	2.2%	0%	0%	0%	0%
	Head deformity	2.2%	0%	0%	1.7%	0%
Group: DEET						
Concentration (mg L^−1^)		325	150	69	31.5	14.5
Sublethality parameters	Pericardial edema	22.2%	0%	0%	2.1%	0%
	Yolk sac edema	22.2%	0%	0%	2.1%	0%
	Spine deformity	0%	4.1%	0%	0%	0%
Group: HPβCD–DEET						
Concentration (mg L^−1^)		C5	C4	C3	C2	C1
Sublethality parameters	Pericardial edema	—	2.6%	1.8%	0%	1.8%
	Yolk sac edema	—	2.6%	1.8%	0%	1.8%
	Tail deformity	—	2.6%	0%	0%	1.8%

—: not determined; all larvae were dead.

According to a literature review by Gould and Scott,^50^ HPβCD is an alternative to α, β, and γCD with improved water solubility as previously mentioned and it also may be safer, since HPβCD is well tolerated in several mammalian species (rats, mice, and dogs), particularly when dosed orally. Maes and coworkers^51^ evaluated a range of HPβCD concentrations (from 0.5% to 2.5%) in zebrafish embryos and larvae for 168 h. The authors also revealed that no developmental stage of this model organism was affected by HPβCD concentrations up to 1% (10 000 mg L^−1^).

**Figure 5:**
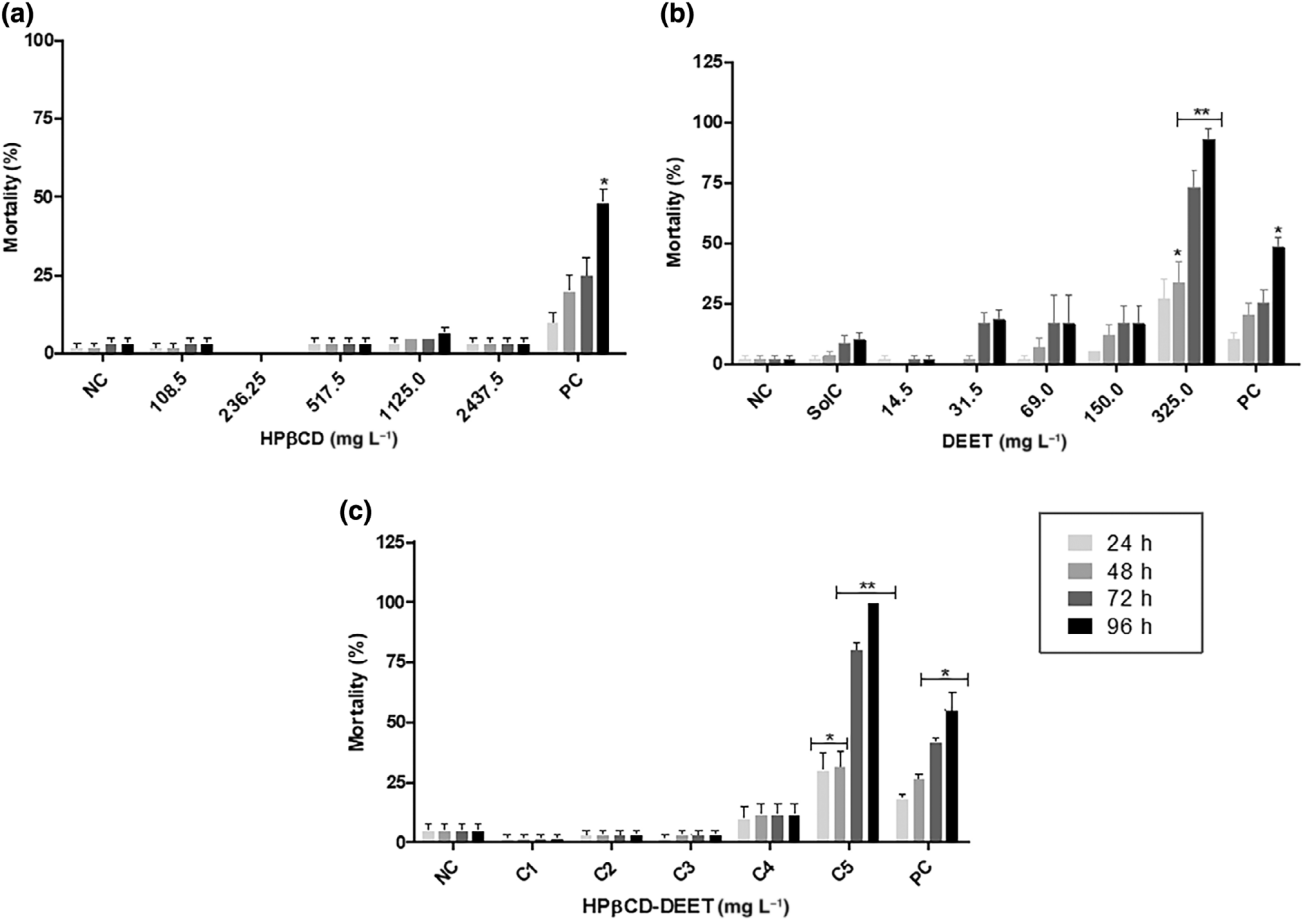
Mortality rate of zebrafish at different developmental stages exposed to (A) HPβCD, (B) DEET, and (C) HPβCD–DEET^#^, during 96 h of exposure. Twenty fertilized eggs per experimental group were evaluated. Bars represent the mean ± standard error of the mean of three independent experiments. *P < 0.05 and **P < 0.01 are statistically different from the respective negative control (NC) based on one‐way ANOVA and Dunnett's *post hoc* test. NC, maintenance water; SolC, DMSO at 0.01%; PC, 3,4‐dichloroaniline at 4 mg L^−1^; C1, 108.5 mg L^−1^ HPβCD + 14.5 mg L^−1^ DEET; C2, 236.25 mg L^−1^ HPβCD + 31.5 mg L^−1^ DEET; C3, 517.5 mg L^−1^ HPβCD + 69 mg L^−1^ DEET; C4, 1125 mg L^−1^ HPβCD + 150 mg L^−1^ DEET; C5, 2437.5 mg L^−1^ HPβCD + 325 mg L^−1^ DEET.

**Figure 6:**
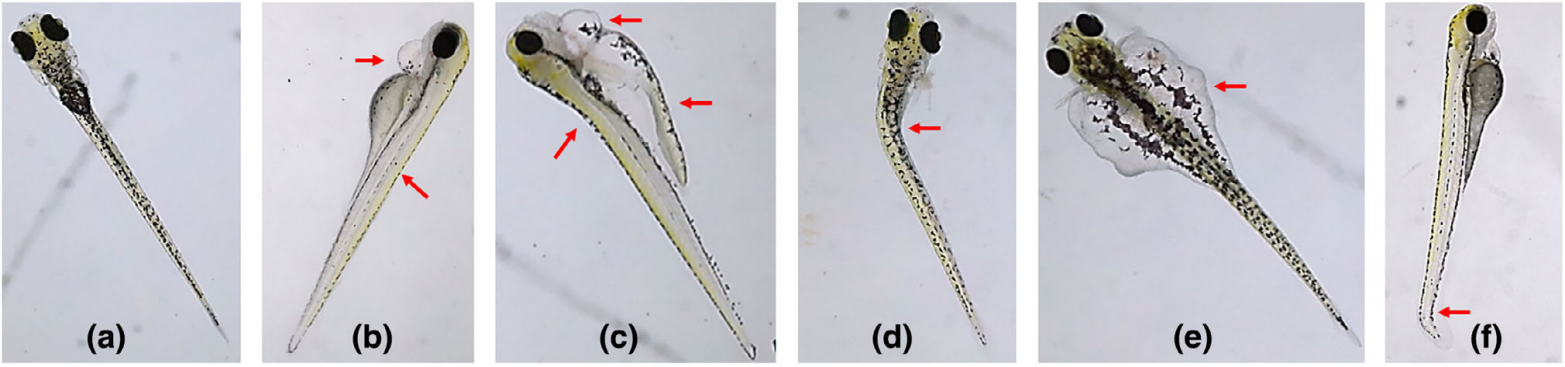
Representative photomicrographs illustrating morphological alterations in zebrafish larvae after 96 h of exposure to HPβCD, DEET, and HPβCD–DEET complex. (A) Negative control (NC; maintenance water) with normal development. (B) HPβCD at 2437.5 mg L^−1^ with pericardial edema. (C) DEET at 150 mg L^−1^ with pericardial and yolk sac edemas and spine deformity. (D) DEET at 69 mg L^−1^ with spine deformity. (E) DEET at 150 mg L^−1^ with yolk sac edema. (F) HPβCD‐DEET at C4 (1125 mg L^−1^ HPβCD + 150 mg L^−1^ DEET) with tail deformity. None of the observed developmental abnormalities were statistically significant.

In relation to DEET, a time‐ and concentration‐dependent lethal effect was observed in embryos and larvae (Fig. 5(B)) with significant mortality occurring only at the highest concentration tested (325 mg L^−1^) from 48 h of exposure. The NC and SolC groups (water and DMSO, respectively) showed survival rates greater than 90%, in accordance with the validation criteria of the FET test. DEET induced some abnormalities, including pericardial and yolk sac edemas, and spine deformity in zebrafish larvae after 96 h of exposure; however, these effects were not statistically significant compared to the NC (Table 2 and Fig. 6), even at the highest tested concentration (325 mg L^−1^).

Based on the literature, DEET is considered safe for use in children aged 2–12 years when applied in concentrations of up to 10%. However, pregnant women and infants younger than 6 months should avoid exposure to DEET‐containing products because of its relative toxicity.^8^ In this study, we assessed the embryotoxicity of DEET based on permeated concentration (69 mg L^−1^) derived from HPβCD–DEET complexes prepared by dispersing 8% DEET in aqueous solutions containing 60% HPβCD. This permeated concentration (69 mg L^−1^) did not induce significant lethal or sublethal effects in zebrafish embryos during 96 h of exposure. As shown, the NOEC and LC_50_ values for DEET were 150 and 201.6 mg L^−1^, respectively (Table 1).

HPβCD–DEET complex (Fig. 5(C)) caused significant embryotoxicity only at the highest concentration tested (C5: 2437.5 mg L^−1^ HPβCD + 325 mg L^−1^ DEET) with lethal effects observed from 24 h (Fig. 5C). This response is likely attributable to the intrinsic toxicity of DEET at elevated concentrations (325 mg L^−1^). Similarly, a low frequency (1.8–2.6%) of abnormalities, such as pericardial and yolk sac edemas and tail deformity, was observed in larvae exposed to the HPβCD–DEET complex. These findings indicate that the formulation is not embryotoxic at DEET concentrations up to 150 mg L^−1^, with a NOEC established at C4 (1125 mg L^−1^ HPβCD + 150 mg L^−1^ DEET) (Table 2).

Regulatory agencies are responsible for protecting human health and the environment by implementing a range of processes to achieve this goal. According to the United States Environmental Protection Agency (EPA), insect repellents are classified as pesticides and therefore certain toxicity tests are required before registering a pesticide product such as acute, subchronic and chronic toxicity testing, developmental and reproductive testing, and mutagenicity testing.^52^

Toxicological studies of formulations are essential for assessing product safety. One study analyzed reports of adverse effects associated with DEET‐containing products in the United States, collected through a post‐marketing surveillance system (National Registry of Human Exposure to DEET, or DEET Registry), identified both severe and mild symptoms. Notably, 36% of cases occurred in children under 13 years of age, and data from the DEET Registry identified seizures as the most commonly reported symptom in this group.^53^

The EPA's Office of Pesticide Programs is developing and evaluating new approach methodologies (NAMs), defined as any technology, methodology, approach, or combination that can provide information on chemical hazard and risk assessment to supplement or replace traditional chemical testing method, such as animal testing, with the goal of better protecting human health and the environment.^52^ In this context, we employed zebrafish as an alternative to traditional mammalian models toxicology studies. This model can substantially increase experimental throughput while reducing costs. Moreover, it offers greater physiological relevance than cell‐based assays, as evaluations are conducted in a whole organism, where complex pharmacokinetic and pharmacodynamic processes are preserved.^54^

Therefore, the zebrafish embryo–larval stage represents a promising NAM for assessing the potential for human prenatal developmental toxicity. With features like external fertilization, transparent development, a high degree of genetic and physiological similarity to humans, many teratogenic effects observed in zebrafish early‐life stages parallel those seen in human congenital abnormalities, making this model a powerful tool for preclinical developmental screening.^55^, ^56^

In summary, our results demonstrate that HPβCD–DEET complex at C3 (517.5 mg L^−1^ HPβCD + 69 mg L^−1^ DEET) did not induce embryotoxicity at the permeated DEET concentration (Fig. 5C and Table 2). Notably, significant lethal effects were observed only at the highest concentration tested (C5: 2437.5 mg L^−1^ HPβCD + 325 mg L^−1^ DEET), where the DEET concentration was approximately 4.7‐fold greater than the amount permeated through the skin in permeation studies. This finding highlights the safety of the CD carrier as a promising alternative for enhancing the solubility of DEET at non‐toxic levels, while also improving its delivery in repellent formulations, providing greater safety to the product.

### 3.4 Repellence laboratorial bioassays

The bioassay for repellency against *A. sculptum* nymphs was performed (Table 3). The percentage of repellency observed for up to 4 h of study was greater than 90%. However, it is interesting to note that HPβCD–DEET complexes repelled *A. sculptum* nymphs for up to 168 h, with effectiveness ranging from 80.5 to 100%, showing a statistically significant (*P* < 0,05) difference at all evaluated times compared to the control. According to Barrozo and coworkers,^7^ these results can be considered as a strong repellency of the formulation against ticks.

**Table 3:** Percentage of repellency (mean ± SD) of unfed *A. sculptum* nymphs exposed to different solutions and formulations in a modified Petri dish assay, evaluated over time intervals ranging from 1 min to 168 h

Time	HPβCD	8% DEET hydroalcoholic solution	HPβCD‐DEET
1′	52.8 ± 12.5	47.2 ± 16.4	94.4 ± 8.6*
3′	50.0 ± 14.9	47.2 ± 16.4	97.2 ± 6.8*
5′	52.8 ± 19.5	52.8 ± 19.5	97.2 ± 6.8*
10′	61.1 ± 27.2*	47.2 ± 16.4	97.2 ± 6.8*
15′	50.0 ± 10.5	47.2 ± 19.5	97.2 ± 6.7*
30′	44.4 ± 17.2	55.6 ± 13.6	94.4 ± 8.6*
60′	52.8 ± 12.5	58.3 ± 20.4	94.4 ± 8.6*
120′	44.4 ± 13.6	61.1 ± 13.6*	91.7 ± 13.9*
240′	41.7 ± 13.9	66.7 ± 21.1*	91.7 ± 9.1*
24 h	30.5 ± 12.5	66.7 ± 10.5	88.8 ± 8.6*
48 h	41.7 ± 17.5*	55.6 ± 25.0	94.4 ± 8.6*
72 h	44.4 ± 20.2*	55.6 ± 8.6	80.5 ± 16.4*
96 h	41.7 ± 17.5	55.6 ± 8.6	83.3 ± 14.9*
168 h	38.9 ± 8.6*	61.1 ± 20.1	80.5 ± 16.4*

Repellency was considered significant when the number of ticks on the control side of the Petri dish was higher than on the treated side, according to the chi‐square test. The tested solutions and formulations were: 60% HPβCD in aqueous solution, 8% DEET in hydroalcoholic solution (ethanol:water 60:40), and HPβCD–DEET (containing 8% DEET).

* Statistically significant difference (*P* < 0.05).

A global mean repellency of 94.4 ± 5.9% was observed on the treated side of Petri dishes with the formulation containing HPβCD–DEET complexes. In contrast, the negative control side, containing only an aqueous solution of HPβCD, showed no significant repellency, with a mean of 44.4 ± 5.9%, that is, it presented a homogeneous distribution, with values close to 50% of ticks on each side of Petri dish. The solutions containing 8% DEET in hydroalcoholic solution exhibited a mean repellency of 55.6 ± 4.8%. Repellency results between 40% and 60% for the control are consistent with values found in the literature^7^, ^45^ and are considered within the expected range. It can be inferred, therefore, that a repellency average of 50% reflects low effectiveness, as the same number of ticks would be equally distributed between the treated and control sides of the Petri dish.

Several studies in the literature report favorable results regarding the efficacy of DEET in repelling ticks. *A. sculptum* has demonstrated a high susceptibility to DEET.^57^ Thus, when DEET is incorporated into a formulation that allows for slow release, the duration of protection can be significantly extended.

The repellency efficacy bioassay against *A. aegypti* under laboratory conditions is shown in Table 4. As observed, the HPβCD–DEET complex provided complete protection for 7 h, maintaining the protection level of >95%, meeting one of the three parameters used in cage tests.^58^ The complex achieved a mean repellency percentage of 98.38 ± 1.67% during the 8 h test, with no statistical difference (*P* > 0.05) compared to the mean repellency percentage of the 8% DEET hydroalcoholic solution.

**Table 4:** Repellency percentage (mean ± SD) of the HPβCD–DEET and the hydroalcoholic DEET solution (ethanol:water, 60:40) on *A. aegypti* mosquitoes in an arm‐in‐the‐cage test over a period of 30 min to 8 h

Time (h)	HPβCD–DEET	8% DEET hydroalcoholic solution
0.5	100.0 ± 0.0	100.0 ± 0.0
1.0	100.0 ± 0.0	100.0 ± 0.0
2.0	100.0 ± 0.0	100.0 ± 0.0
3.0	100.0 ± 0.0	100.0 ± 0.0
4.0	100.0 ± 0.0	100.0 ± 0.0
5.0	98.0 ± 2.8	100.0 ± 0.0
6.0	98.1 ± 2.7	100.0 ± 0.0
7.0	95.8 ± 1.1	100.0 ± 0.0
8.0	93.6 ± 4.4	100.0 ± 0.0

Arthropod repellents can effectively deter ticks, mosquitoes, and other hematophagous arthropods from biting humans and thus transmitting pathogens.^59^ When comparing the results of the repellency bioassays, the inclusion complex tested in this study, HPβCD–DEET containing 8% DEET, resulted in a high repellency percentage comparable to ticks and mosquitoes.

## 4 CONCLUSION

Our results show that HPβCD–DEET complexes, under the proposed conditions, can promote a slow release of DEET over time, increasing both retention of the active ingredient within the formulation and its effectiveness compared to formulations containing conventional drug solubilization systems, such as organic solvents. In a laboratory study, the general repellent efficacy during 168 h remained higher than 90% for *A. sculptum*. The repellency efficacy bioassay against *A. aegypti* provided complete protection for 7 h, maintaining the protection level of >95%. Furthermore, CDs are essentially non‐toxic, and the low levels of DEET permeated showed no toxicity, which makes this system very promising.

## CONFLICT OF INTEREST

The authors declare no conflicts of interest.

## AUTHOR CONTRIBUTIONS

RNM and SFT: Conceptualization, funding acquisition, project administration, supervision, writing – review and editing. JR, GARdeO, CM, CL and JR: Supervision, validation, writing – review and editing. GRSP, GC, MLKC, MRM, MMB and JR: Data curation, formal analysis, investigation, validation, methodology. GRSP, GC, JR, CM and CL: Investigation, resources and writing – original draft.

## Supporting information


**Figure S1.** *In vitro* repellency assay against *A. sculptum* nymphs. (a) Petri dish assembly for laboratory repellence study. (b) Schematic representation of the repellency test.
**Figure S2.** *In vivo* repellent assay against *Aedes aegypti* mosquitoes. (a) Cage used in the experiments, designed according to WHO specifications (dimensions 30 × 20 × 20 cm), containing 36 adult female mosquitoes [46]. (b) *In vivo* repellency test — insertion of the volunteer's untreated control arm, with a defined exposure area (3 × 10 cm) delimited using a plastic stopper and a rubber glove, showing mosquito landings on the exposed untreated skin.

## Data Availability

The data that support the findings of this study are available from the corresponding author upon reasonable request.
