# Supplementary material for: DEET‐cyclodextrin inclusion complexes for mosquito and tick repellency: Extended repellency time and no zebrafish embryotoxicity
Source: Pest Manag Sci. 2026 Jun 11;82(10):9653–65. doi: 10.1002/ps.71001 (PMC13569254; doi:10.1002/ps.71001)
Supplement: Supplementary file 1 [file PS-82-9653-s001.docx]

**Supplementary Material**

**DEET-cyclodextrin inclusion complexes for mosquito and tick repellency: extended repellency time and embryotoxicity studies in zebrafish model**

Gessyka Rayana Silva Pereira^a^, Gleidson Cardoso^a^, Maria Luiza Kuhn Camargo^a^, Michele Resende Machado^b^, Mayara Macedo Barrozo^c^, Juscelino Rodrigues^d^, Gisele Augusto Rodrigues de Oliveira^b^, Caio Monteiro^c^, Christian Luz^d^, Ricardo Neves Marreto^a^ and Stephânia Fleury Taveira^a^*.

^a^Laboratory of Nanosystems and Drug Delivery Devices (NanoSYS), School of Pharmacy, Universidade Federal de Goiás (UFG). Rua 240, esq. com 5ª Avenida, Setor Leste Universitário. 74605-170, Goiânia – GO, Brazil.

^b^Enviromental Toxicology Research Laboratory (Envtox), School of Pharmacy, Universidade Federal de Goiás (UFG). Rua 240, esq. com 5ª Avenida, Setor Leste Universitário. 74605-170, Goiânia – GO, Brazil.

^c^Biology, Ecology and Tick Control Laboratory (LaBEC), Veterinary and Animal Science School, Universidade Federal de Goiás (UFG). Rodovia Goiânia, km 8 s/n Campus – Samambaia. 74001-970, Goiânia – GO, Brazil.

^d^Laboratory of Parasitology, Instituto de Patologia Tropical e Saúde Pública (IPTSP), Universidade Federal de Goiás (UFG). R. 235, s/n.º, Setor Leste Universitário, Goiânia – GO, Brazil.

*Correspondence: Prof. Stephânia Fleury Taveira. E-mail: stephaniafleury@ufg.br. Tel/Fax: +55 62 3209-6519.

**Figure S1.** In vitro repellency assay against A. sculptum nymphs: (a) petri dish assembly for laboratory repellence study, (b) schematic representation of the repellency test.

(a) **(b)**

**Figure S2.** *In vivo* repellent assay against *Aedes aegypti* mosquitoes: (a) Cage used in the experiments, designed according to WHO specifications (dimensions: 30 × 20 × 20 cm), containing 36 adult female mosquitoes^46^; (b) *In vivo* repellency test — insertion of the volunteer’s untreated control arm, with a defined exposure area (3 × 10 cm) delimited using a plastic stopper and a rubber glove, showing mosquito landings on the exposed untreated skin.

(a) **(b)**
